# Identification of Motivational Determinants for Telemedicine Use Among Patients With Rheumatoid Arthritis in Germany: Secondary Analysis of Data From a Nationwide Cross-Sectional Survey Study

**DOI:** 10.2196/47733

**Published:** 2024-08-19

**Authors:** Felix Muehlensiepen, Pascal Petit, Johannes Knitza, Martin Welcker, Nicolas Vuillerme

**Affiliations:** 1 Center for Health Services Research Faculty of Health Sciences Brandenburg Brandenburg Medical School Theodor Fontane Rüdersdorf bei Berlin Germany; 2 AGEIS Université Grenoble Alpes Grenoble France; 3 Department of Internal Medicine 3 Friedrich-Alexander-University Erlangen-Nürnberg and Universitätsklinikum Erlangen Erlangen Germany; 4 Institute for Digital Medicine, University Hospital Giessen-Marburg Philipps University Marburg Marburg Germany; 5 Medizinisches Versorgungszentrum für Rheumatologie Dr M Welcker GmbH Planegg Germany; 6 Institut Universitaire de France Paris France; 7 LabCom Telecom4Health, Orange Labs & Université Grenoble Alpes, CNRS, Inria Grenoble France

**Keywords:** telemedicine, rheumatoid arthritis, rheumatology, primary care, health services research, eHealth, data analysis, survey, Germany, tool, care, willingness, sociodemographic, age, telehealth, digital transition

## Abstract

**Background:**

Previous studies have demonstrated telemedicine to be an effective tool to complement rheumatology care and address workforce shortage. With the COVID-19 outbreak, telemedicine experienced a massive upswing. An earlier analysis revealed that the motivation of patients with rheumatic and musculoskeletal diseases to use telemedicine is closely connected to their disease. It remains unclear which factors are associated with patients’ motivation to use telemedicine in certain rheumatic and musculoskeletal diseases groups, such as rheumatoid arthritis (RA).

**Objective:**

This study aims to identify factors that determine the willingness to try telemedicine among patients diagnosed with RA.

**Methods:**

We conducted a secondary analysis of data from a German nationwide cross-sectional survey among patients with RA. Bayesian univariate logistic regression analysis was applied to the data to determine which factors were associated with willingness to try telemedicine. Predictor variables (covariates) studied individually included sociodemographic factors (eg, age, sex) and health characteristics (eg, health status). All the variables positively and negatively associated with willingness to try telemedicine in the univariate analyses were then considered for Bayesian model averaging analysis after a selection based on the variance inflation factor (≤ 2.5) to identify determinants of willingness to try telemedicine.

**Results:**

Among 438 surveyed patients in the initial study, 210 were diagnosed with RA (47.9%). Among them, 146 (69.5%) answered either yes or no regarding willingness to try telemedicine and were included in the analysis. A total of 22 variables (22/55, 40%) were associated with willingness to try telemedicine (region of practical equivalence %≤5). A total of 9 determinant factors were identified using Bayesian model averaging analysis. Positive determinants included desiring telemedicine services provided by a rheumatologist (odds ratio [OR] 13.7, 95% CI 5.55-38.3), having prior knowledge of telemedicine (OR 2.91, 95% CI 1.46-6.28), residing in a town (OR 2.91, 95% CI 1.21-7.79) or city (OR 0.56, 95% CI 0.23-1.27), and perceiving one’s health status as moderate (OR 1.87, 95% CI 0.94-3.63). Negative determinants included the lack of an electronic device (OR 0.1, 95% CI 0.01-0.62), absence of home internet access (OR 0.1, 95% CI 0.02-0.39), self-assessment of health status as bad (OR 0.44, 95% CI 0.21-0.89) or very bad (OR 0.47, 95% CI 0.06-2.06), and being aged between 60 and 69 years (OR 0.48, 95% CI 0.22-1.04) or older than 70 years (OR 0.38, 95% CI 0.16-0.85).

**Conclusions:**

The results suggest that some patients with RA will not have access to telemedicine without further support. Older patients, those not living in towns, those without adequate internet access, reporting a bad health status, and those not owning electronic devices might be excluded from the digital transformation in rheumatology and might not have access to adequate RA care. These patient groups certainly require support for the use of digital rheumatology care.

## Introduction

Rheumatoid arthritis (RA) is a chronic inflammatory joint disease, which can cause cartilage and bone damage as well as disability [1]. RA is the most common rheumatic disease with a global age-standardized point prevalence and annual incidence rates of 246.6 and 14.9 per 100,000 population in 2017 [2]. RA is associated with high medical costs [3] and contributes to a significant deterioration in quality of life [4]. Patients in rural areas usually have limited access to rheumatology care and therefore, accept longer diagnosis times [5]. The increasing shortage of rheumatologists alongside rising demand makes RA a major global public health challenge [2].

Telemedicine offers a promising opportunity to support RA care [6,7]. It has the potential to address workforce shortage [8] as well as disparity in rheumatology care for rural patients [4]. The effective integration of telemedicine into standard rheumatology care, depends on the willingness and ability of end users to engage with telemedicine [9,10].

A previous study revealed that patients’ motivation to use telemedicine is closely connected to their specific disease [11]. However, it is still unclear which factors determine the motivation of patients with RA for using telemedicine. To gain a better understanding of these factors, we performed a secondary analysis using data from a German nationwide cross-sectional survey conducted earlier [10]. The aim of this study was to identify the factors associated with RA patients’ willingness to try telemedicine.

## Methods

### Study Design

This work reports on results from a secondary analysis of data collected as part of a cross-sectional, self-completed, and paper-based survey of German patients with rheumatic and musculoskeletal diseases (RMDs) in collaboration with the patient organization German League against Rheumatism (Deutsche Rheuma-Liga, Landesvertretung Brandenburg) and outpatient rheumatologists. The eligibility criteria to participate in the survey were being (1) a patient in rheumatology care; (2) aged 18 years and older; and (3) in Germany. The survey was embedded in a more than 2-year mixed methods study, investigating acceptance, opportunities, and obstacles to the implementation of telemedicine [10]. The actual survey was conducted between September 1, 2019, and December 30, 2019. The exact methodology applied for the survey has been described previously [10].

### Data Selection or Population Considered

From the aforementioned German nationwide survey, a data set included 438 patients in total, only 210 (47.9%) patients were diagnosed with RA and were considered in this study. These patients answered a total of 26 questions (Table 1) [11]. Individuals with missing answer regarding willingness to try telemedicine or that answered, “do not know” (Q11: “Would you like to try telemedicine?”) were excluded from this study. As a result, a total of 146 (146/210, 69.5%) patients were considered for analysis.

To check if this sample size was enough to answer the goal of our study, we conducted a sample size estimation based on a 1-sample proportion test aiming at determining whether the proportion of individuals who wanted to try telemedicine was significantly different from participants who did not want to try telemedicine. Due to the lack of similar studies regarding this topic, the sample size estimation was based on conservative measures. To that end, we considered a significant level of .05, a statistical power of 0.80, and a small effect size (h=0.25) [12], resulting to an estimated sample size of 126. The choice of this size effect value was motivated by a previous study [11]. In this previous study, there was a response rate of 71.7% regarding the willingness to try telemedicine among patients, with 116 of 282 patients (41.1%) who were willing to try telemedicine. Assuming that a similar proportion of patients is willing to try telemedicine and a similar response rate among the subsample of patients diagnosed with RA, the corresponding Cohen *h* (effect size) would be 2asin(sqrt(0.41))–2asin(sqrt(0.410.717))=0.244≈0.25, which corresponds to a medium effect size [12-14]. Using the exact Cohen *h* value would have yielded an estimated sample size of 132.

To address the selection bias introduced by excluding individuals with missing answer regarding willingness to try telemedicine or that answered, “do not know,” a comparison of the characteristics of included and excluded participants was performed (Multimedia Appendix 1). To that end, a Wilcoxon-Mann-Whitney rank sum test was performed for continuous variables, while the chi-square test for count data was used for categorical variables. The Wilcoxon effect size and its 95% CI were computed to quantify the difference between both groups using the Vargha and Delaney *A* statistic [15]. To measure the strength of the association between categorical variables, the Cramér *V* was computed, with a value of 1 corresponding to complete association and of 0 corresponding to no association between the variables.

**Table 1:** Regression analysis—variables considered (n=146).

				Modality		Response rate, n (%)
**Dependent variable**						
		Q11: “Would you like to try telemedicine?”	2 categories: yes or no		146 (100)	
**Independent variables**						
	Q1: “How far do you drive to your rheumatology doctor’s office?”			6 categories: up to 10 km, 10-20 km, 20-30 km, 30-40 km, >40 km, not answered		145 (99.3)
	Q2: “How far do you drive to your general practitioner’s office?”			5 categories: up to 5 km, 5-10 km, 10-15 km, >15 km, not answered		146 (100)
	Q3: “Have you ever contacted your doctor’s office using an electronic means?”			3 categories: yes, no, not answered		146 (100)
	Q4: “Do you own an electronic device?”			3 categories: yes, no, not answered		145 (99.3)
	Q5: “Do you have internet access at home?”			3 categories: yes, no, not answered		146 (100)
	Q8: “Prior to this survey, had you ever heard the term ‘telemedicine’?”			4 categories: yes, no, do not know, not answered		145 (99.3)
	Q14: “Would you like your rheumatologist offer you telemedicine services?”			4 categories: yes, no, do not know, not answered		139 (95.2)
	Q16: “Do you document your health status?”			4 categories: yes on paper, yes digitally, no, not answered		139 (95.2)
	Q17: Age			4 categories: <60 years, 60-69 years, >70 years, not answered		144 (98.6)
	Q18: Biological sex			3 categories: female, male, not answered		144 (98.6)
	Q20: “How do you rate your health status?”			6 categories: very good, good, moderate, bad, very bad, not answered		141 (96.6)
	Q21: “Are you in rheumatology treatment?”			4 categories: yes, no I am a new patient, do not know, not answered		146 (100)
	Q23: “My place of residence is…”			5 categories: city (>100,000 inhabitants), town (20,000-100,000 inhabitants), provincial town (5000-20,000 inhabitants), rural area (<5000 inhabitants), not answered		144 (98.6)

### Choice of Variables

Questions related to other RMDs were not considered in this study, leaving a total of 14 questions (Table 1). “Would you like to try telemedicine?” (Q11) was used as dependent variable while age (Q17), biological sex (Q18), distance to the rheumatologist’s office (Q1), distance to the GP’s office (Q2), previous electronic contact with physician (Q3), electronic device ownership (Q4), internet access (Q5), self-reported knowledge regarding telemedicine (Q8), self-reported willingness that rheumatologist offer telemedicine services (Q16), self-reported health status (Q20), means of health status documentation (Q16), rheumatology treatment (Q21), and place of residence (Q23) were used as independent variables. For questions other than willingness to try telemedicine (Q11: “Would you like to try telemedicine?”), missing values (no answer) were considered as a new category for the regression analysis [16]. For instance, Q4 (“Do you own an electronic device?”) had previously 2 categories and was considered with 3 (yes, no, and not answered). As a result, 55 answers from 13 independent variables were considered (Table 1). For statistical analysis, all the categorical variables having more than 2 modalities (eg, “yes,” “no,” “do not know”) were transformed into dummy or binary variables. For instance, Q4 (“Do you own an electronic device?”) was transformed into 3 dummy variables. Age was considered as both continuous and categorical variables.

### Regression Analysis

Bayesian univariate logistic regression analyses was applied to the data in order to determine which factors were associated with willingness to try telemedicine (Q11: “Would you like to try telemedicine?”).

For each model, odds ratios (ORs) with 95% CI are presented. All the individual variables associated (positively or negatively) with willingness to try telemedicine in the Bayesian univariate analysis were considered for analysis in later Bayesian multivariate analysis after variable selection. This variable selection was based on the ROPE (region of practical equivalence) percentage (ROPE%≤ 5) [17-19], and a subsequent selection based on the variance inflation factor (VIF) [20]. Collinear covariates, with a VIF>2.5, were excluded in the multivariate models [21]. Finally, determinants of willingness to try telemedicine were identified through Bayesian model averaging (BMA) [22].

All statistical analyses were performed using R software (version 4.1.2; R Foundation for Statistical Computing) for Windows 10. The sample size estimation was performed using the *pwr* package version 1.3-0 [13]. Bayesian estimation was performed using the *rstanarm* package version 2.21.1 [23,24]. Weakly informative priors (default prior in *rstanarm*) were used. The default priors in *rstanarm* package are designed to be weakly informative. The Bayesian model adds priors (independent by default) on the coefficients of the generalized linear model. The Bayesian estimation was performed via Markov chain Monte Carlo Bernoulli model, with 4 randomly initialized Markov chains, each for 2000 iterations (including a warm-up period of 1000 iterations that is discarded). The BMA was undertaken with the *BMA* package version 3.18.15 [25]. Regarding priors for BMA, we assumed that all candidate models were equally likely a priori (same prior weight).

### Ethical Considerations

Primary data collection was conducted in compliance with the current data protection regulations of the General Data Protection Regulation [26] and the Helsinki Declaration. All the study participants were informed about the research project. Sending the questionnaire back to the study center was considered as consent. No personal data was collected in the survey; therefore, no written informed consent was required in accordance with the General Data Protection Regulation [26]. Participants did not receive any compensation for participating in this study. The institutional review board of the Theodor Fontane Medical School in Brandenburg issued a waiver for the secondary analyses of the survey data (164112023-ANF).

## Results

### Population Characteristics

The response rate from the 146 patients with RA included in this study varied from 95.2% (139/146) for Q14 (“Would you like your rheumatologist offer you telemedicine services?”) and Q16 (“Do you document your health status?”) to 100% (146) for Q2 (“How far do you drive to your general practitioner’s office?”), Q3 (“Have you ever contacted your doctor’s office using an electronic means?”), Q5 (“Do you have internet access at home?”), and Q21 (“Are you in rheumatology treatment?”), respectively (Table 1). Most patients with RA were female (111/146, 76.0%), with an average age of 62 (SD 13.2) years and lived in provincial town or rural area (86/146, 58.9%; Table 2). Half of the patients with RA considered they had bad or very bad health status (70/146, 47.9%), while 41.1% (60/146) considered their health status as moderate. Most patients with RA had internet access at home (120/146, 82.2%) and owned an electronic device (131/146, 89.7%) while half of the patients had prior telemedicine knowledge (79/146, 54.1%).

To address potential selection bias, we compared the characteristics of included (146/210, 69.5%) and excluded (64/210, 30.5%) participants (Multimedia Appendix 1). The mean age was similar between excluded (60, SD 15) years and included participants (62, SD 13) years, with no significant difference (*P*=.46). Similar proportions of included (79/146, 54.1%) and excluded (34/64, 53%) participants had prior knowledge of telemedicine, with no significant difference (*P*=.98). There were no significant differences in the distance to the rheumatologist’s or general practitioner’s office between included and excluded participants (*P*=.28 and *P*=.69, respectively). Similarly, no significant differences were found in terms of electronic contact with physicians (*P*=.48), health status (*P*=.60), rheumatology treatment (*P*=.20), or place of residence (*P*=.36). A slightly higher proportion of included participants had internet access at home (120/146, 82.1% vs 47/63, 73%), but this difference was not significant (*P*=.21). There were no significant differences in health status documentation methods (*P*=.27) or sex distribution (*P*=.31) between the groups. Most participants in both groups owned an electronic device, with no significant difference (*P*=.94). Yet, a higher proportion of included participants answered yes or no regarding their wish that telemedicine services were offered by their rheumatologist (116/146, 79.4%) than excluded participants (30/63, 48%), with a higher proportion of included participants who wished for telemedicine services offered by their rheumatologist (33/146, 22.6% vs 8/63, 12%) than excluded participants (*P*<.001). Because we excluded participants who answered “do not know” regarding willingness to try telemedicine (Q11), there was, by construction, a significant difference between excluded and included participants regarding Q11.

**Table 2:** Characteristics of the study population^a^.

Question			All participants (n=146)		Participants who answered yes to willingness to try telemedicine (Q11)		Participants who answered no to willingness to try telemedicine (Q11)		Chi-square (*df*)			*P* value			Cramer *V*	
**Want to try TM (Q11), n (%)**										140 (1)			1.2 x 10^–32^			0.98
	Yes, n (%)	51 (34.9)		51 (100)		0 (0)										
	No, n (%)	95 (65.1)		0 (0)		95 (100)										
Age, mean (SD)			62 (13)		65 (12)		57 (15)		3183^b^			7.0 x 10^–4^			0.67 (0.58-0.75)^c^	
**Prior knowledge of TM (Q8), n (%)**										8.4 (2)			.02			0.24
	Yes	79 (54.1)		36 (71)		43 (45)										
	No	63 (43.1)		14 (27)		49 (52)										
	Do not know	3 (2.1)		1 (2)		2 (2)										
	Missing answer or not answered	1 (0.68)		0 (0)		1 (1)										
**Distance to rheumatologist’s office (Q1), n (%)**										3.6 (6)			.74			0.16
	Up to 10 km	52 (35.6)		19 (3)		33 (35)										
	10-20 km	27 (18.5)		8 (16)		19 (20)										
	20-30 km	23 (15.7)		7 (14)		16 (17)										
	30-40 km	18 (12.3)		9 (18)		9 (10)										
	40-50 km	14 (9.6)		3 (6)		11 (12)										
	50-60 km	3 (2.1)		1 (2)		2 (2)										
	More than 60 km	8 (5.5)		3 (6)		5 (5)										
	Missing answer or not answered	1 (0.68)		1 (2)		0 (0)										
**General practitioner’s office distance (Q2), n (%)**										6.4 (5)			.26			0.21
	Up to 5 km	103 (70.5)		33 (65)		70 (74)										
	5-10 km	22 (15.1)		9 (18)		13 (14)										
	10-15 km	11 (7.5)		4 (8)		7 (7)										
	15-20 km	7 (4.8)		2 (4)		5 (5)										
	20-25 km	1 (0.68)		1 (2)		0 (0)										
	25-30 km	2 (1.4)		2 (4)		0 (0)										
	>30 km	0 (0)		0 (0)		0 (0)										
**Electronic contact with physician (Q3), n (%)**										1.9 (1)			.16			0.11
	No	34 (23.2)		8 (16)		26 (27)										
	Yes	112 (76.7)		43 (84)		69 (73)										
	Missing answer or not answered	0 (0)		0 (0)		0 (0)										
**Health status (Q20), n (%)**										7.5 (4)			.11			0.23
	Very good	3 (2.1)		2 (4)		1 (1.1)										
	Good	8 (5.5)		4 (8)		4 (4)										
	Moderate	60 (41.1)		26 (51)		34 (36)										
	Bad	61 (41.7)		15 (29)		46 (48)										
	Very bad	9 (6.2)		2 (4)		7 (7)										
	Missing answer or not answered	5 (3.4)		2 (4)		3 (3)										
**Rheumatology treatment (Q21)**																
	Yes	139 (95.2)		47 (92)		92 (97)		3.9 (2)			.14			0.16		
	No, I am a new patient	5 (3.4)		2 (4)		3 (3)										
	Do not know	2 (1.4)		2 (4)		0 (0)										
**Place of residence (Q23), n (%)**										6.1 (3)			.11			0.21
	City (>100,000 inhabitants)	35 (23.9)		9 (18)		26 (27)										
	Town (20,000-100,000 inhabitants)	23 (15.7)		13 (25)		10 (11)										
	Provincial town (5000-20,000 inhabitants)	46 (31.5)		16 (31)		30 (32)										
	Rural area (< 5000 inhabitants)	40 (27.4)		13 (25)		27 (28)										
	Missing answer or not answered	2 (1.4)		0 (0)		2 (2)										
**Internet access at home (Q5), n (%)**										8.9 (1)			2.8 x 10^–3^			0.25
	Yes	120 (82.2)		49 (96)		71 (75)										
	No	26 (17.8)		2 (4)		24 (25)										
**Wish of telemedicine services offered by rheumatologist (Q14), n (%)**										56 (2)			8.8 x 10^–13^			0.63
	Yes	33 (22.6)		26 (51)		7 (7)										
	No	83 (56.8)		8 (16)		75 (79)										
	Do not know	23 (15.8)		12 (24)		11 (12)										
	Missing answer or not answered	7 (4.8)		5 (10)		2 (2)										
**Health status documentation (Q16), n (%)**										0.88 (2)			.64			0.08
	Yes, on paper	35 (23.9)		13 (25)		22 (23)										
	Yes digitally	19 (13.0)		8 (16)		11 (12)										
	No	85 (58.2)		27 (53)		58 (61)										
	Missing answer or not answered	7 (4.8)		3 (6)		4 (4)										
**Sex (Q18), n (%)**										0 (1)			>.99			0
	Male	33 (22.6)		12 (24)		21 (22)										
	Female	111 (76.0)		39 (76)		72 (76)										
	Missing answer or not answered	2 (1.4)		0 (0)		2 (2)										
**Possession of an electronic device (Q4), n (%)**										4.1 (1)			.04			0.17
	Yes	131 (89.7)		50 (98)		81 (85)										
	No	14 (9.6)		1 (2)		13 (14)										
	Missing answer or not answered	1 (0.68)		0 (0)		1 (1)										

^a^All variables are reported as number (percentage), except for age (mean, SD). Wilcoxon-Mann-Whitney rank sum test for age and chi-square test for count data was used to compare participants’ characteristics between those who answered yes and no.

^b^*W* statistic.

^c^Vargha and Delaney *A* statistic.

### Bayesian Univariate Logistic Regression Analysis

**Figure 1:**
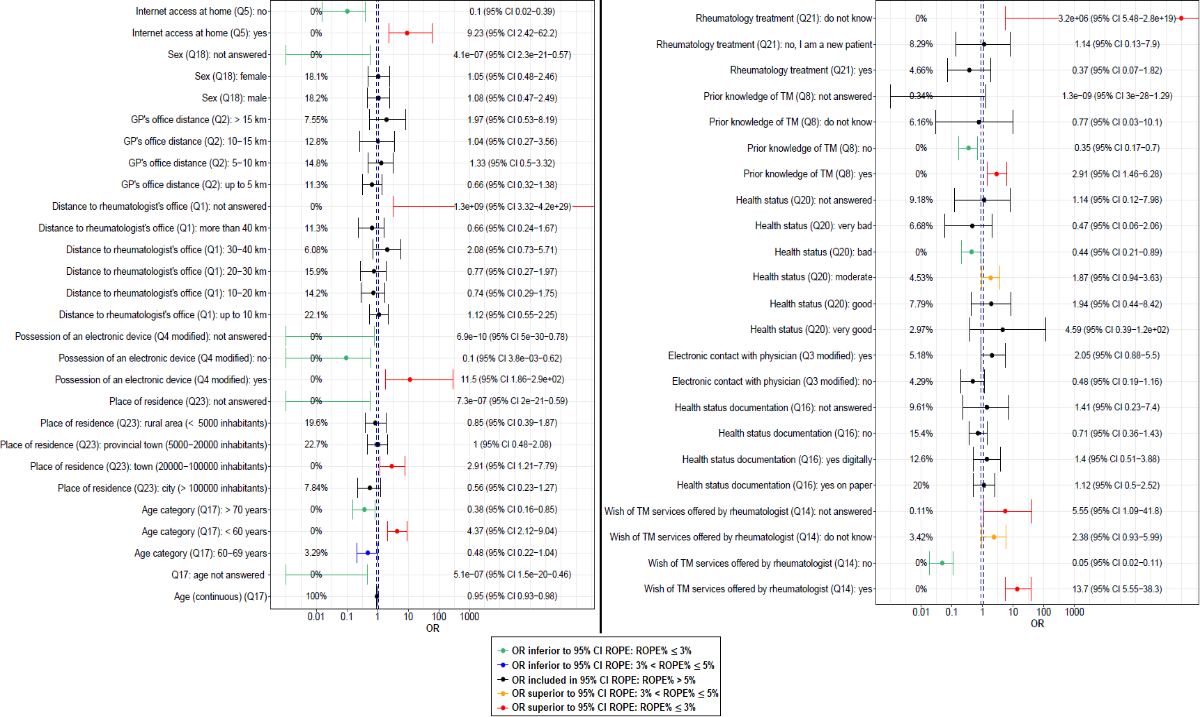
Bayesian univariate logistic regression. The percentage indicates ROPE, that is, the probability that the considered credible factor values are not negligible. The dashed lines indicate the 95% CI of the ROPE. ROPE: region of practical equivalence.

A total of 22 (40%) out of 55 variables or factors (answers to the 13 questions) were found to be positively or negatively associated (ROPE%≤5) with willingness to try telemedicine (Figure 1). After removing collinear variables (VIF>2.5), a total of 9 (40%) out of 22 variables were considered for the BMA analysis.

### BMA Analysis

**Figure 2:**
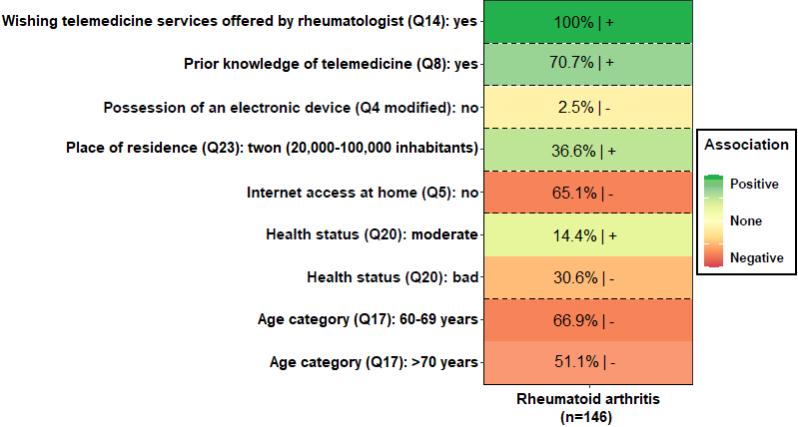
Determinants of willingness to try telemedicine identified through Bayesian model averaging analysis.

Figure 2 presents the factors identified through BMA. The value in each cell corresponds to the posterior probability that the considered variable is nonzero (in percent). The darker the color, the higher the posterior probability percentage. Cells with color from light yellow to red and the “+” sign refer to factors positively associated with willingness to try telemedicine. By contrast, cells with colors from light green to dark blue and the “–” sign refer to factors negatively associated with willingness to try telemedicine. A total of 8 determinant factors were identified. Wishing that telemedicine services were offered by a rheumatologist, possessing prior knowledge of telemedicine, living in a town and perceiving one’s health status as moderate were the 4 factors positively associated with willingness to try telemedicine. By contrast, not owning an electronic device, not having home internet access, perceiving one’s health status as bad and being over 60 years were the 4 factors negatively associated with willingness to try telemedicine.

**Figure 3:**
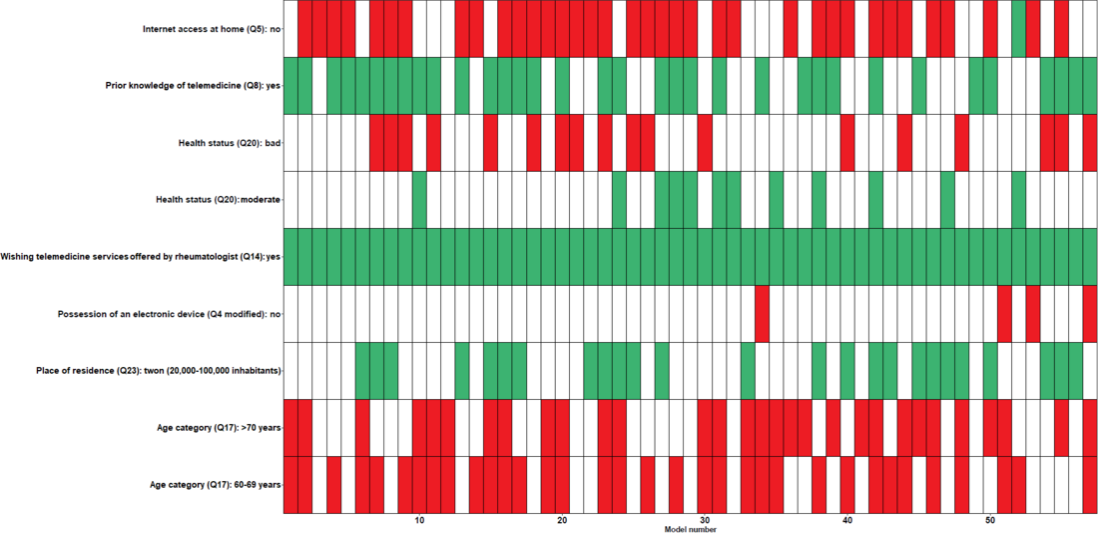
Bayesian model averaging analysis—factors selected for each candidate model tested. Green cells refer to variables that were positively associated with willingness to try telemedicine while red cells indicate variables that are negatively associated with willingness to try telemedicine.

Figure 3 presents the detailed results of the BMA analysis, which tests multiple plausible or candidate models to explain willingness to try telemedicine based on the observed data. A total of 57 models were tested (x-axis), with the independent variables selected (y-axis) for each model depicted with colored cells. For a given model, green cells refer to independent variables that were positively associated with willingness to try telemedicine, while red cells indicate independent variables that are negatively associated with willingness to try telemedicine.

### Patients’ Profile

**Figure 4:**
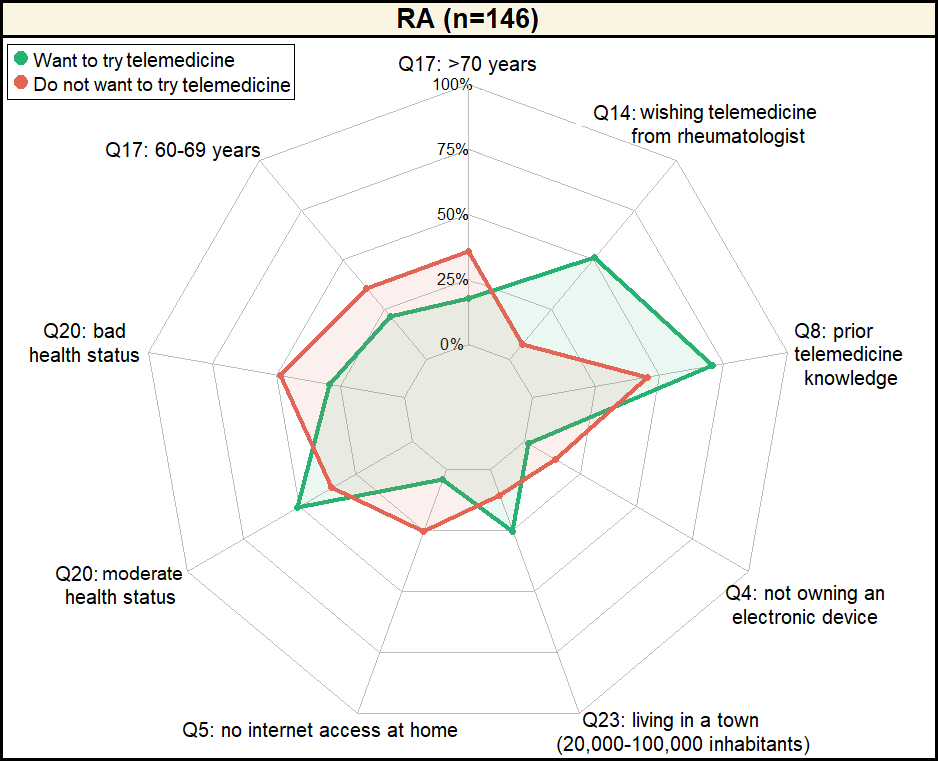
Profile of patients with RA motivated to try telemedicine versus patients with RA not motivated to try telemedicine. The percentages refer to the percentage of individuals with the answer specified for each question. RA: rheumatoid arthritis.

The BMA analysis delineated 2 distinct profiles of RA patients based on their motivation toward telemedicine (Figure 4). Figure 4 shows the profiles identified with patients who are motivated to try telemedicine highlighted in green and patients who do not want to try telemedicine highlighted in red.

Older patients with RA, particularly those aged over 60 years, demonstrated less inclination toward telemedicine, with 72% (68/95) showing reluctance, in contrast to 39% (20/51) of the more motivated younger cohort. This motivation disparity extends to health perceptions, where 48% (46/95) of the telemedicine-reluctant group reported poor health status, compared to 29% (15/51) among those keen on telemedicine.

A closer examination of technological engagement reveals that patients with a positive attitude toward telemedicine possess a stronger foundation in digital literacy, evidenced by 70% (36/51) having prior telemedicine knowledge and 96.1% (49/51) having internet access at home. This group also boasts a higher rate of electronic device ownership (50/51, 98%) and a marked preference for living in urban areas (13/51, 25%). Interestingly, these patients perceive their health status more moderately than their counterparts.

The data underscores a strong demand among telemedicine-enthusiastic patients for the integration of telemedicine services by their rheumatologists, with 51% (26/51) expressing a wish for such offerings, a stark contrast to the 7% (7/95) among the less motivated group. This segmentation of patients with RA by telemedicine motivation offers insightful parameters for tailoring telehealth services to meet diverse patient needs and preferences effectively.

The percentage indicates the ROPE percentage, that is, the probability that the considered credible factor values are not negligible. The dashed lines indicate the 95% CI of the ROPE.

The percentages refer to the percentage of patients with RA with the answer specified for each question according to the patient motivation to try telemedicine or not (ie, patients with RA who want to try telemedicine and patients with RA who do not want to try telemedicine). For instance, 100% (the outer circular line, the farthest from the radar center) means that all patients with RA have answered the considered question with the specified answer (eg, being 60-69 years). By contrast, 0% (the inner circular line the closest to the center) means that no patient with RA chose the specified answer for the considered question (eg, being greater than 70 years). The points indicate for each question the percentage of patients with RA that chose the specified answer. Green points and lines refer to patients that wanted to try telemedicine. Red points and lines correspond to patients not wanting to try telemedicine. For each question, there were 3 possible situations. First, when the green and red points overlapped (were similar), it meant that there was no difference between patients with RA whether they were motivated or not to use telemedicine, the proportion of similar answers was close. Second, when the green point was higher (higher percentage) than the red point, it indicates that patients with RA motivated to use telemedicine chose the specified answer more often than patients with RA not motivated to use telemedicine, which means that this factor (answer to the question) had a positive impact on willingness to try telemedicine. Finally, when the green point was lower (lower percentage) than the red point, it indicates that patients with RA motivated to use telemedicine chose the specified answer less often than patients with RA not motivated to use telemedicine, which means that this factor (answer to the question) had a negative impact on willingness to try telemedicine.

## Discussion

### Overview

The effective use of telemedicine in RA care offers the highest potential for improving health outcomes and self-management of patients along with relieving rheumatology workforce and reducing health care costs [27-29]. Therefore, we conducted a secondary analysis using data from a German nationwide cross-sectional survey among patients with RMD [11] to identify the factors associated with the willingness to try telemedicine, aiming to enable more effective telemedicine strategies.

### Principal Results

Our results revealed that factors determining the motivation of patients with RA toward telemedicine use were multidimensional.

On one hand, patients with RA who wanted to try telemedicine more often owned an electronic device and had internet access at home. They were less likely to be younger than 60 years, perceived their health condition as moderate, had prior knowledge of traditional medicine, and more often lived in a city or town.

On the other hand, patients with RA not wanting to try telemedicine, had less access to internet at home, no prior knowledge of telemedicine and considered to have a bad health status.

### Comparison With Prior Work

Only limited research findings on the effectiveness and acceptability of telemedicine approaches in rheumatology have been published prior to the beginning of the COVID-19 pandemic [7,30,31]. However, our results are consistent with previous studies: Müskens et al [27] reported that the Dutch users of a telemedicine platform were younger, more highly educated, and had better health outcomes than the total population with RA. Concurrently, our data confirms a demographic divide in the use of eHealth and mobile health: patients with RA aged less than 60 years were willing to try telemedicine, whereas those aged 60 to 80 years did not want their rheumatologists to offer them telemedicine. Furthermore, similar to the corresponding findings among physicians [32], the knowledge of telemedicine was an important determinant of telemedicine motivation among patients with RA. Our results are consistent with previous studies. Indeed, Hansen et al [33] revealed a digital divide in terms of education level in the use of eHealth platforms (apps, search engines, video services, and social media sites) among people with diabetes in Norway. Tennant et al [34] showed that being younger, using more electronic devices, and possessing a higher level of education positively influences eHealth literacy among residents of the US state of Florida. The perceived health status is also a key determinant in terms of telemedicine motivation among patients with RA. Patients, who considered their health status to be bad rarely wanted to use telemedicine, whereas patients with a moderate health status were interested in telemedicine. We hypothesize here that the worse the disease state, the higher the desire to talk face-to-face with a rheumatologist. In contrast, Ferrucci et al [35] reported that video telemedicine was more likely to be used by Alaskan patients with RA with higher disease activity. In conclusion, the relationship between disease state and telemedicine use needs to be further explored.

Complementing the demographic and health status divide, we also found signs of an economic divide in telemedicine motivation. Our results emphasize that the individual possession of technical, telemedicine-ready, equipment determines the motivation for usage. Those patients with RA who do not own technical devices will not use telemedicine. These findings are in line with previous studies for other diseases pointing to the digital divide and the danger of socioeconomic inequalities in the use of eHealth. Based on data of National Cancer Institute’s 2012 Health Information National Trends Survey, Kontos et al [36] identified that that lower socioeconomic status, older, and male US adult internet-users were less likely to engage in a number of eHealth activities, such as using the internet to look for a health care provider, using email or the internet to communicate with a doctor, using web-based tracking of their personal health information, using a website to help track diet, weight, and physical activity, or downloading health information to a smartphone device. Latulippe et al [37] recently performed a literature review with the result that ethnicity and low income are the most commonly used characteristics to identify people at risk of social health inequality, which might be reduced via eHealth by aiming for universal access to the tool of eHealth, considering of users’ literacy level, creating eHealth tools that respect the cultural attributes of future users, and encouraging the participation of people at risk of social health inequalities. Since educational level, socioeconomic status, ethnicity, and income were not assessed in the original survey, there is an urgent need for further research to gain a better understanding of access to telemedicine in rheumatology.

### Implications

In the realm of rheumatology, the implementation of telemedicine presents both opportunities and challenges. Our findings underscore the necessity of ensuring that all patients with RA can access telemedicine, highlighting the importance of addressing barriers related to age, geographical location, internet connectivity, and the availability of electronic devices. To circumvent these obstacles, targeted support and educational initiatives are imperative. Programs aimed at patient education, bolstered by the active involvement of patient associations, have been identified as crucial factors in facilitating the adoption of telehealth among patients with RA [38]. Such measures not only promote digital literacy but also ensure that patients are not excluded from receiving comprehensive RA care due to technological disparities.

The clinical practice landscape is similarly affected, with physicians identified as key facilitators in the adoption of telemedicine [39]. However, the uptake of telemedicine by health care professional is hindered by challenges mirroring those faced by patients, including a lack of familiarity with telemedicine, demographic factors such as age, and the geographical context of their practice [40]. Overcoming these barriers is essential to broaden the use of telemedicine in clinical settings. Proposals such as the integration of assisted telemedicine services in pharmacies or health kiosks, particularly in rural and underserved regions, are promising strategies that align with broader digitalization efforts in Germany [41]. These initiatives can play a pivotal role in enhancing access to specialized rheumatology care, ensuring that digital health resources extend their reach effectively.

Moreover, the evolution of medical teaching to incorporate telemedicine knowledge is critical. The education of health care professionals must prioritize digital literacy, ensuring that future clinicians are proficient in using digital tools for patient care. This includes a comprehensive understanding of how to navigate the challenges and opportunities presented by telemedicine. Furthermore, as telemedicine becomes increasingly integrated into standard health care delivery, it is paramount to maintain the option of high-quality, traditional, and analogue health care. This approach necessitates the implementation of a triage mechanism [42] to match patients with the most suitable form of care, whether digital or traditional, tailored to their preferences and circumstances.

### Limitations

The primary data on which this analysis is based was collected until December 30, 2019, that is, shortly prior to SARS-CoV-2 outbreak in Germany (January 27, 2020). Due to the need to reduce physical contact and thus minimize the risk of infection, usage of telemedicine initially received a major uptake in global health care delivery [43]. Hence, more patients with RMDs and likely other subgroups will have tried telemedicine by now [44]. A replication of the initial survey is essential to identify whether and how the identified factors have changed. Apart from this, the limitations of the primary data still apply [10]. These are primarily the high potential of self-selection and nonresponse bias. These sources of bias are due to the fact that the primary data collection was a questionnaire survey in which participation was voluntary. These biases may have contributed to a disproportionate representation of technically proficient and interested patients in the survey population, potentially leading to a more favorable attitude toward telemedicine. Additionally, the study presents a selection bias because participants who either did not answer Q11 (“Would you like to try telemedicine?”) or answered “do not know” were not included in our analysis, possibly excluding those who are not familiar with the term “telemedicine.” However, this selection bias should be limited because the characteristics of the excluded individuals were similar to those of the included participants (Multimedia Appendix 1). To mitigate this limited selection bias, a multinomial regression analysis, considering all participants, regardless of their answer, could have been performed. Furthermore, it is important to emphasize that there is a considerable difference between the inclination expressed in a survey and the actual use of telemedicine by patients with RMD. Our data must be validated against real-world data from clinical practice in RA care.

Regarding statistical analyses, we used a Bayesian approach to conduct the secondary analysis of the earlier survey. A practical limitation of the Bayesian approach is that it requires the specification of prior distributions both on parameters of each model and on the distribution of models themselves. As we had no a priori assumption, we used weakly informative priors. Choosing another prior distribution may have substantial influence on the outcome [45,46]. Regarding variable selection, a widespread approach consisting of including significant variables from univariate analysis in a multivariate analysis was carried out [47,48]. To be more accurate, all the individual variables associated (positively or negatively) with willingness to try telemedicine in the Bayesian univariate analysis were selected based on the ROPE percentage (ROPE %≤5). A ROPE-only decision rule was used as suggested in other works [16-18]. Choosing another ROPE percentage threshold may have yielded different results. Then, we performed a conservative selection based on the VIF (≤2.5) to deal with potential variable multicollinearity. Finally, we used the remaining variables with BMA for model selection and identification of determinants. BMA was chosen in particular because it reduces overconfidence and is relatively robust against model misspecification [46,49-51]. The Markov chain Monte Carlo model was used to deal with the intractable computational challenge of BMA that comes from the candidate model enumeration [52].

### Conclusions

Our results suggest that health status, telemedicine knowledge, age, and access to technical equipment and infrastructure influence the motivation of patients with RA to use telehealth. In particular, older patients with RA with bad health status, are currently not motivated to use telemedicine and, thus, might be left out from digital transition in rheumatology care.

## Appendix

Multimedia Appendix 1 Comparison of the characteristics (continuous variable) of the included and excluded participants.

## Abbreviations

BMA: Bayesian model averaging
OR: odds ratio
RA: rheumatoid arthritis
RMD: rheumatic and musculoskeletal disease
ROPE: region of practical equivalence
VIF: variance inflation factor

## Data Availability

The data sets generated during or analyzed during this study are available from the corresponding author on reasonable request.
