# Supplementary material for: Identification of Motivational Determinants for Telemedicine Use Among Patients With Rheumatoid Arthritis in Germany: Secondary Analysis of Data From a Nationwide Cross-Sectional Survey Study
Source: J Med Internet Res. 2024 Aug 19;26:e47733. doi: 10.2196/47733 (PMC11369527; doi:10.2196/47733)
Supplement: Multimedia Appendix 1 [file jmir_v26i1e47733_app1.docx]

**Table S1. Comparison of the characteristics (continuous variable) of the included and excluded participants.**

| **Characteristics** | **Excluded participants (n=64)** | | | | **Included participants (n=146)** | | | | **W** | **p** | **VDA** |
| --- | --- | --- | --- | --- | --- | --- | --- | --- | --- | --- | --- |
|  | n (NA%) | mean ± sd | median [IQR] | [min-max] | n (NA%) | mean ± sd | median [IQR] | [min-max] |  |  |  |
| Age | 64 (0) | 60 ± 15 | 63 [19] | [21-79] | 146 (1.4) | 62 ± 13 | 64 [16] | [22-85] | 4309 | 0.46 | 0.47 [0.38; 0.55] |

*Note: IQR: interquartile range, max: maximum, min: minimum, NA: missing value, sd: arithmetic standard deviation, VDA: Vargha and Delaney’s A statistic.*

**Table S2. Comparison of the characteristics (categorical variable) of the included and excluded participants.**

| **Question** | **Excluded participants (n=64)** | **Included participants (n=146)** | **Chi2** | **P** | **Cramer's V** |
| --- | --- | --- | --- | --- | --- |
| Prior knowledge of TM (Q8): yes | 34 (53%) | 79 (54%) | 0.05 | 0.98 | 0.02 |
| Prior knowledge of TM (Q8): no | 27 (42%) | 63 (43%) | 0.05 | 0.98 | 0.02 |
| Prior knowledge of TM (Q8): do not know | 1 (1.6%) | 3 (2.1%) | 0.05 | 0.98 | 0.02 |
| Prior knowledge of TM (Q8): not answered | 2 (3.1%) | 1 (0.68%) | 0.05 | 0.98 | 0.02 |
| Want to try TM (Q11): missing answer or do not know | 64 (100%) | 0 (0%) | 210 | **2.5e-46** | 1 |
| Want to try TM (Q11): yes | 0 (0%) | 51 (35%) | 210 | **2.5e-46** | 1 |
| Want to try TM (Q11): no | 0 (0%) | 95 (65%) | 210 | **2.5e-46** | 1 |
| Distance to rheumatologist's office (Q1): up to 10 km | 21 (33%) | 52 (36%) | 7.5 | 0.28 | 0.19 |
| Distance to rheumatologist's office (Q1): 10-20 km | 8 (12%) | 27 (18%) | 7.5 | 0.28 | 0.19 |
| Distance to rheumatologist's office (Q1): 20-30 km | 13 (20%) | 23 (16%) | 7.5 | 0.28 | 0.19 |
| Distance to rheumatologist's office (Q1): 30-40 km | 9 (14%) | 18 (12%) | 7.5 | 0.28 | 0.19 |
| Distance to rheumatologist's office (Q1): 40-50 km | 2 (3.1%) | 14 (9.6%) | 7.5 | 0.28 | 0.19 |
| Distance to rheumatologist's office (Q1): 50-60 km | 4 (6.2%) | 3 (2.1%) | 7.5 | 0.28 | 0.19 |
| Distance to rheumatologist's office (Q1): more than 60 km | 6 (9.4%) | 8 (5.5%) | 7.5 | 0.28 | 0.19 |
| Distance to rheumatologist's office (Q1): not answered | 1 (1.6%) | 1 (0.68%) | 7.5 | 0.28 | 0.19 |
| GP's office distance (Q2): up to 5 km | 46 (72%) | 103 (71%) | 3.9 | 0.69 | 0.14 |
| GP's office distance (Q2): 5-10 km | 10 (16%) | 22 (15%) | 3.9 | 0.69 | 0.14 |
| GP's office distance (Q2): 10-15 km | 5 (7.8%) | 11 (7.5%) | 3.9 | 0.69 | 0.14 |
| GP's office distance (Q2): 15-20 km | 2 (3.1%) | 7 (4.8%) | 3.9 | 0.69 | 0.14 |
| GP's office distance (Q2): 20-25 km | 0 (0%) | 1 (0.68%) | 3.9 | 0.69 | 0.14 |
| GP's office distance (Q2): 25-30 km | 0 (0%) | 2 (1.4%) | 3.9 | 0.69 | 0.14 |
| GP's office distance (Q2): >30 km | 1 (1.6%) | 0 (0%) | 3.9 | 0.69 | 0.14 |
| Electronic contact with physician (Q3): no | 18 (28%) | 34 (23%) | 0.49 | 0.48 | 0.05 |
| Electronic contact with physician (Q3): yes | 44 (69%) | 112 (77%) | 0.49 | 0.48 | 0.05 |
| Electronic contact with physician (Q3): not answered | 2 (3.1%) | 0 (0%) | 0.49 | 0.48 | 0.05 |
| Health status (Q20): very good | 2 (3.1%) | 3 (2.1%) | 2.8 | 0.6 | 0.12 |
| Health status (Q20): good | 1 (1.6%) | 8 (5.5%) | 2.8 | 0.6 | 0.12 |
| Health status (Q20): moderate | 23 (36%) | 60 (41%) | 2.8 | 0.6 | 0.12 |
| Health status (Q20): bad | 32 (50%) | 61 (42%) | 2.8 | 0.6 | 0.12 |
| Health status (Q20): very bad | 4 (6.2%) | 9 (6.2%) | 2.8 | 0.6 | 0.12 |
| Health status (Q20): not answered | 2 (3.1%) | 5 (3.4%) | 2.8 | 0.6 | 0.12 |
| Rheumatology treatment (Q21): yes | 64 (100%) | 139 (95%) | 3.2 | 0.2 | 0.12 |
| Rheumatology treatment (Q21): no, I am a new patient | 0 (0%) | 5 (3.4%) | 3.2 | 0.2 | 0.12 |
| Rheumatology treatment (Q21): do not know | 0 (0%) | 2 (1.4%) | 3.2 | 0.2 | 0.12 |
| Place of residence (Q23): city (>100000 inh.) | 9 (14%) | 35 (24%) | 3.2 | 0.36 | 0.13 |
| Place of residence (Q23): town (20000-100000 inh.) | 10 (16%) | 23 (16%) | 3.2 | 0.36 | 0.13 |
| Place of residence (Q23): provincial town (5000-20000 inh.) | 19 (30%) | 46 (32%) | 3.2 | 0.36 | 0.13 |
| Place of residence (Q23): rural area (< 5000 inh.) | 23 (36%) | 40 (27%) | 3.2 | 0.36 | 0.13 |
| Place of residence (Q23): not answered | 3 (4.7%) | 2 (1.4%) | 3.2 | 0.36 | 0.13 |
| Internet access at home (Q5): yes | 47 (73%) | 120 (82%) | 1.6 | 0.21 | 0.09 |
| Internet access at home (Q5): no | 17 (27%) | 26 (18%) | 1.6 | 0.21 | 0.09 |
| Wish of TM services offered by rheumatologist (Q14): yes | 8 (12%) | 33 (23%) | 20 | **4.0e-05** | 0.32 |
| Wish of TM services offered by rheumatologist (Q14): no | 22 (34%) | 83 (57%) | 20 | **4.0e-05** | 0.32 |
| Wish of TM services offered by rheumatologist (Q14): do not know | 27 (42%) | 23 (16%) | 20 | **4.0e-05** | 0.32 |
| Wish of TM services offered by rheumatologist (Q14): not answered | 7 (11%) | 7 (4.8%) | 20 | **4.0e-05** | 0.32 |
| Health status documentation (Q16): yes on paper | 14 (22%) | 35 (24%) | 2.6 | 0.27 | 0.12 |
| Health status documentation (Q16): yes digitally | 3 (4.7%) | 19 (13%) | 2.6 | 0.27 | 0.12 |
| Health status documentation (Q16): no | 37 (58%) | 85 (58%) | 2.6 | 0.27 | 0.12 |
| Health status documentation (Q16): not answered | 10 (16%) | 7 (4.8%) | 2.6 | 0.27 | 0.12 |
| Sex (Q18): male | 10 (16%) | 33 (23%) | 1 | 0.31 | 0.07 |
| Sex (Q18): female | 54 (84%) | 111 (76%) | 1 | 0.31 | 0.07 |
| Sex (Q18): not answered | 0 (0%) | 2 (1.4%) | 1 | 0.31 | 0.07 |
| Possession of an electronic device (Q4): yes | 56 (88%) | 131 (90%) | 4.9e-03 | 0.94 | 4.8e-03 |
| Possession of an electronic device (Q4): no | 7 (11%) | 14 (9.6%) | 4.9e-03 | 0.94 | 4.8e-03 |
| Possession of an electronic device (Q4): not answered | 1 (1.6%) | 1 (0.68%) | 4.9e-03 | 0.94 | 4.8e-03 |

*Note: inh.: inhabitants.*
